# Supplementary material for: Systematic genetic analysis of the MHC region reveals mechanistic underpinnings of HLA type associations with disease
Source: eLife. 2019 Nov 20;8:e48476. doi: 10.7554/eLife.48476 (PMC6904215; doi:10.7554/eLife.48476)
Supplement: Transparent reporting form [file elife-48476-transrepform.pdf]

eLIFE
1st Floor
24 Hills Road
Cambridge CB2 1JP, UK
P 01223 855340
W elifesciences.org
T @elife

eLife's transparent reporting form

We encourage authors to provide detailed information *within their submission* to facilitate the interpretation and replication of experiments. Authors can upload supporting documentation to indicate the use of appropriate reporting guidelines for health-related research (see EQUATOR Network), life science research (see the BioSharing Information Resource), or the ARRIVE guidelines for reporting work involving animal research. Where applicable, authors should refer to any relevant reporting standards documents in this form.

If you have any questions, please consult our Journal Policies and/or contact us: editorial@elifesciences.org.

Sample-size estimation

- You should state whether an appropriate sample size was computed when the study was being designed
- You should state the statistical method of sample size computation and any required assumptions
- If no explicit power analysis was used, you should describe how you decided what sample (replicate) size (number) to use

Please outline where this information can be found within the submission (e.g., sections or figure legends), or explain why this information doesn't apply to your submission:

The analyses performed in this manuscript use publicly available data: we used the two largest collections of iPSC lines with whole genome sequence data. One of these collections (HipSci) had 146 individuals and the other (iPSCORE) had 273. In terms of WGS, these are the largest collections of samples genotyped for HLA genes at 8-digit resolution. For the eQTL analysis, the size of our collection (361 RNA-seq samples) is comparable to most GTEx tissues.

Replicates

- You should report how often each experiment was performed
- You should include a definition of biological versus technical replication
- The data obtained should be provided and sufficient information should be provided to indicate the number of independent biological and/or technical replicates
- If you encountered any outliers, you should describe how these were handled
- Criteria for exclusion/inclusion of data should be clearly stated
- High-throughput sequence data should be uploaded before submission, with a private link for reviewers provided (these are available from both GEO and ArrayExpress)

Please outline where this information can be found within the submission (e.g., sections or figure legends), or explain why this information doesn't apply to your submission:

Biological replicates (twin pairs and matched iPSC-fibroblast samples) were used to calculate recall rate and accuracy of calling HLA genes at 8 digit resolution (Figure 1C-E).
No outlier data was removed from this study.
Data is publicly available through dbGaP and ENA. Additional details on the study IDs are provided in the Methods section.
Summary statistics are available through Figshare.

Statistical reporting

- Statistical analysis methods should be described and justified
- Raw data should be presented in figures whenever informative to do so (typically when N per group is less than 10)
- For each experiment, you should identify the statistical tests used, exact values of N, definitions of center, methods of multiple test correction, and dispersion and precision measures (e.g., mean, median, SD, SEM, confidence intervals; and, for the major substantive results, a measure of effect size (e.g., Pearson's r, Cohen's d)
- Report exact p-values wherever possible alongside the summary statistics and 95% confidence intervals. These should be reported for all key questions and not only when the p-value is less than 0.05.

Please outline where this information can be found within the submission (e.g., sections or figure legends), or explain why this information doesn't apply to your submission:

Statistical analysis methods to call HLA types have been previously published (HLA-VBSeq). For conducting the eQTL analysis we used linear mixed models from (lmekin) from the coxme package in R.
Raw data is provided in supplemental tables. Summary statistics are available through Figshare.
No analyses in this study use groups with N < 10.
Statistical tests are described in detail in the main text, figures and supplemental tables and exact p-values are reported.

(For large datasets, or papers with a very large number of statistical tests, you may upload a single table file with tests, Ns, etc., with reference to sections in the manuscript.)

Group allocation

- Indicate how samples were allocated into experimental groups (in the case of clinical studies, please specify allocation to treatment method); if randomization was used, please also state if restricted randomization was applied
- Indicate if masking was used during group allocation, data collection and/or data analysis

Please outline where this information can be found within the submission (e.g., sections or figure legends), or explain why this information doesn't apply to your submission:

Samples from iPSCORE and HipSci were treated as distinct groups only for examining WGS read depth. All other analyses were performed on these two datasets merged.

Additional data files ("source data")

- We encourage you to upload relevant additional data files, such as numerical data that are represented as a graph in a figure, or as a summary table
- Where provided, these should be in the most useful format, and they can be uploaded as "Source data" files linked to a main figure or table
- Include model definition files including the full list of parameters used
- Include code used for data analysis (e.g., R, MatLab)
- Avoid stating that data files are "available upon request"

Please indicate the figures or tables for which source data files have been provided:

Sequence data is publicly available in dbGaP and ENA. HLA genotypes and eQTL results are provided in supplemental tables. All statistical analyses were performed using publicly available R code as described in the Methods section.

eLife Sciences Publications, Ltd is a limited liability non-profit non-stock corporation incorporated in the State of Delaware, USA, with company number 5030732, and is registered in the UK with company number FC030576 and branch number BR015634 at the address 1st Floor, 24 Hills Road, Cambridge CB2 1JP | August 2014
